# Gelsolin as a Potential Clinical Biomarker in Psoriasis Vulgaris

**DOI:** 10.3390/jcm12051801

**Published:** 2023-02-23

**Authors:** Sul Hee Lee, Young-Lip Park, Youin Bae

**Affiliations:** 1 Department of Dermatology, Soonchunhyang University Bucheon Hospital, Bucheon 14584, Republic of Korea; 2 Department of Dermatology, College of Medicine, Hallym University, Chuncheon 24252, Republic of Korea; 3 Department of Dermatology, Hallym University Dongtan Sacred Heart Hospital, Hwaseong 18450, Republic of Korea

**Keywords:** gelsolin, mass spectrometry, proteomics, psoriasis vulgaris

## Abstract

Although discovering novel biomarkers for psoriasis is challenging, it may play an essential role in diagnosis, severity assessment, and prediction of treatment outcome and prognosis. The study was aimed to determine potential serum biomarkers of psoriasis via proteomic data analysis and clinical validity assessment. Thirty-one subjects manifested psoriasis and 19 subjects were healthy volunteers who were enrolled in the study. Protein expression was performed via two-dimensional gel electrophoresis (2-DE) using psoriasis patients’ sera before and after treatment and sera of patients without psoriasis. Image analysis was then performed. Nano-scale liquid chromatography-tandem mass spectrometry (LC-MS/MS) experiments subsequently identified points showing differential expression in 2-DE image analysis. To measure levels of candidate proteins to validate results obtained from 2-DE, enzyme linked immunosorbent assay (ELISA) was then conducted. Gelsolin was identified as a potential protein through LC-MS/MS analysis and database search. Serum gelsolin levels were lower in the groups of psoriasis patients before treatment than in the control group and the group of psoriasis patients after treatment. Additionally, in subgroup analysis, serum gelsolin level was correlated with various clinical severity scores. In conclusion, low serum gelsolin levels are associated with the severity of psoriasis, proposing the potential role of gelsolin as a biomarker for severity assessment and evaluation of treatment response of psoriasis.

## 1. Introduction

Psoriasis is a chronic inflammatory, immune-mediated skin disease globally affecting 2–5% of the human population [1]. Psoriasis can have a profound negative impact on the physical, emotional, and quality of life of affected patients [2]. Although the pathogenesis of psoriasis is not completely elucidated yet, advances in understanding of immunological pathways in psoriasis have led to the development of molecular-targeted therapies including biologics and small-molecule inhibitors [3].

In addition to efforts to determine the pathogenesis of psoriasis, efforts are underway to discover reliable biomarkers that can be useful for diagnosing the disease and classifying phenotypes, as well as evaluating the clinical severity and treatment response. To date, clinicians have mainly used and relied on clinical assessment tools such as Psoriasis Area and Severity Index (PASI) and physician global assessment (PGA) to evaluate the severity of psoriasis and therapeutic outcome.

A significant number of genetic, tissue, and serum markers have been identified and utilized in the pathomechanism and diagnosis of psoriasis and evaluation of severity or prediction of treatment response. Among these, human β-defensin, vascular endo thelial growth factor, and lipocalin-2 are known as biomarkers related to disease activity, and markers that may help predict response to drugs including HLA-C 06 and thymus and activation-regulated chemokine [4,5,6,7]. However, the usability of biomarkers discovered to date varies drastically in reflecting the activity or severity of the disease. Moreover, most genetic and tissue biomarkers are not approachable because their measurement and visualization usually require advanced experimental methods. In this context, attempts to identify serum biomarkers that are correlated with psoriasis severity and treatment response based on serum tests are needed. 

Gelsolin is a protein of the gelsolin superfamily. It is encoded by the gelsolin gene located on the human chromosome 9 [8]. It is a highly conserved, multifunctional actin-binding protein first described in the cytosol of macrophages and later in many other cells [9]. This protein is involved in the remodeling of the cytoskeletal structure that determines morphology of cells, chemotaxis, and secretion [10]. Its extracellular secretory isoform, called plasma gelsolin, is presented in various body fluids including blood, cerebrospinal fluid, milk, urine and other extracellular fluids [11,12].

The objective of the study was to conduct proteomic data analysis via two-dimensional gel electrophoresis (2-DE) and nanoscale liquid chromatography-tandem mass spectrometry (LC-MS/MS) to identify differentially expressed proteins between before and after treatment of psoriasis vulgaris (PV). Based on proteomic analysis and subsequent database search, gelsolin was presented as a candidate protein. To validate this protein as a dependable biomarker, serum levels of the gelsolin were measured in serum collects of a larger number of PV patients and controls and compared according to disease severity index. Finally, correlations between clinical severity scores and gelsolin level in pooled serum samples at baseline and post-treatment were determined.

## 2. Materials and Methods

### 2.1. Enrollment of Study Subjects

Patients with PV diagnosed both clinically and histopathologically were enrolled for proteomic analysis and the validation study. Inclusion criteria were: (1) patients diagnosed as chronic plaque type psoriasis for more than 2 years, (2) patients with age more than 18 years, (3) those who had never treated with any systemic or biologic agent in the prior year, and (4) those with a body surface area (BSA) of at least 10% and PASI score of at least 10 who were eligible for biologic treatment according to the British association of dermatologists guidelines [13].

Exclusion criteria were as: (1) patients who underwent major surgery in the prior year, (2) those who had unstable cardiovascular disease or a cardiac hospitalization within prior year, (3) those who had unstable pulmonary disease or a pulmonary hospitalization within the prior year, (4) those had any major illness or evidence of an unstable clinical condition, (5) those who had cancer or history of cancer within the previous 5 years, (6) those who tested positive for interferon-gamma release assay and (7) those who had been diagnosed rheumatoid arthritis or ankylosing spondylitis. 

Participants were treated with oral methotrexate (MTX) or a biologic agent (secukinumab, ixekizumab, guselkumab or risankizumab) for more than 52 weeks. Healthy volunteers without a history of psoriasis or psoriatic arthritis also participated. This study was approved by the Institutional Review Board (IRB) of Hallym University Dongtan Sacred Heart Hospital (IRB file No. HDT 2020-06-025). All participants were fully informed and voluntarily signed the informed consent. 

### 2.2. Clinical and Laboratory Data Collection

Clinical characteristics of participants including age, sex, duration of the PV, and accompanying diseases were collected. Severity scores were assessed with PASI, PGA, and Dermatology Life Quality Index (DLQI) scores before treatment (baseline), at 16 weeks of treatment, and at 52 weeks of treatment. Laboratory data including CBC, routine chemistry were also measured at baseline, at 16 weeks of treatment, and at 52 weeks of treatment.

### 2.3. Blood Sample Collection and Preparation

Blood samples were collected from participants. In addition to conventional venous blood sampling, additional 3 mL samples of blood were collected into a 5 mL BD vacutainer serum separation tubes for proteomic experiment. These tubes were allowed to clot at room temperature for 30 min. They were then centrifuged at 1300× *g* for 10 min in a swinging bucket centrifuge within 2 h. Separated serum was transferred into 1.5 mL tubes in 500 μL aliquots and stored at −70 °C until further use [14].

### 2.4. Two-Dimensional Gel Electrophoresis (2-DE) and Image Analysis

Two-dimensional gel electrophoresis (2-DE) was conducted as reported. Briefly, aliquots in sample buffer (7 M urea, 2 M thiourea, 4.5% CHAPS, 100 mM DTE, 40 mM Tris, pH 8.8) were added to immobilized pH 3–10 nonlinear gradient strips (Amersham Biosciences, Uppsala, Sweden). Isoelectrofocusing (IEF) was performed at 80,000 Vh. The second dimension was analyzed on 9–16% linear gradient polyacrylamide gels (18 cm × 20 cm × 1.5 mm) at a constant 40 mA per gel for approximately 5 h. After protein fixation in 40% methanol and 5% phosphoric acid for 1 h, the gels were stained with CBB G-250 for 12 h. The gels were destained with H_2_O, scanned with a GS710 densitometer (Bio-Rad, Richmond, CA, USA) and converted to electronic files, which were then analyzed with an Image Master Platinum 5.0 image analysis program (Amersham Biosciences, Amersham, UK).

### 2.5. LC-MS/MS Analysis of Peptides

Proteomic analysis via LC-MS/MS technique was performed with an Easy *n*-LC (Thermo Fisher Scientific, San Jose, CA, USA) and a LTQ Orbitrap XL mass spectrometer (Thermo Fisher Scientific, San Jose, CA, USA) equipped with a nano-electrospray source. Serum collects were separated on a C18 nanobore column (150 mm × 0.1 mm, 3 μm pore size; Agilent). The mobile phase A for LC separation included 0.1% formic acid and 3% acetonitrile in deionized water and the mobile phase B was 0.1% formic acid in acetonitrile. The chromatography gradient was designed for a linear increase from 0% B to 60% B in 9min, 60% B to 90% B in 1 min, and 3% B in 5 min. The flow rate was maintained at 1800 nL/min. 

Mass spectra were acquired using data-dependent acquisition with a full mass scan (380–1700 *m*/*z*) followed by 10 MS/MS scans. The orbitrap resolution for MS1 full scans was 15,000 and the automatic gain control (AGC) was 2 × 10^5^. For MS/MS in the LTQ, the AGC was 1 × 10^4^ [14]. 

### 2.6. Database Search

To identify peptide sequences in a protein sequence database, Mascot algorithm (Matrix Science, Boston, MA, USA) was utilized. Database search parameters were: *Homo sapiens*; *Homo sapiens*, fixed modification; carbamidomethylated at cysteine residues, variable modification; oxidized at methionine residues, maximum allowed missed cleavage; 2, MS tolerance; 10 ppm, MS/MS tolerance; 0.8 Da. Peptides were filtered with a significance threshold of *p* < 0.05 [14].

### 2.7. Enzyme Linked Immunosorbent Assay for Validation Study 

Serum levels of candidate protein were measured using enzyme linked immunosorbent assays (ELISA) (Nobus Biologicals, Centennial, CO, USA) in accordance with the manufacturer’s instruction. The resultant color reaction was read at 450nm using a microplate reader. 

### 2.8. Statistical Analysis

Data are expressed as mean ± SD. Statistical analyses were performed using SPSS statistics 21 (IBM SPSS Inc., Chicago, IL, USA). Statistical analyses were executed using Student’s *t*-test, one-way ANOVA followed by multiple comparisons with Tukey’s honestly significant difference test. Pearson correlation analysis was also completed. A *p*-value of less than 0.05 was considered significant.

## 3. Results

### 3.1. Characteristics of Study Subjects

Clinical characteristics of psoriasis patients are listed in Table 1. Initially, 57 participants were enrolled the study. After excluding seven psoriasis patient who reported that they had latent tuberculosis (*n* = 3), rheumatoid arthritis (*n* = 2), or history of cancer (*n* = 2), among a total of fifty participants (30 males and 20 females; mean age of 39.68 ± 11.29 years), thirty-one subjects manifested psoriasis and nineteen subjects were healthy volunteers. The proportion of sex and age showed no significant difference between the two groups. The psoriasis group was divided into pre-treatment (baseline) and two post-treatment subgroups (16 and 52 weeks from baseline). All patients in the psoriasis group received MTX or one of the four biologics (secukinumab, ixekizumab, guselkumab, or risankizumab) for more than 52 weeks. All subjects showed clinical improvement in PASI and PGA scores as shown in Table 1. Of all patients, only three patients failed to achieve PASI 75 in week 52. All three were patients who were taking MTX.

### 3.2. 2-DE and Image Comparison between Control, Pre-Treatment and Post-Treatment Groups

**Figure 1:**
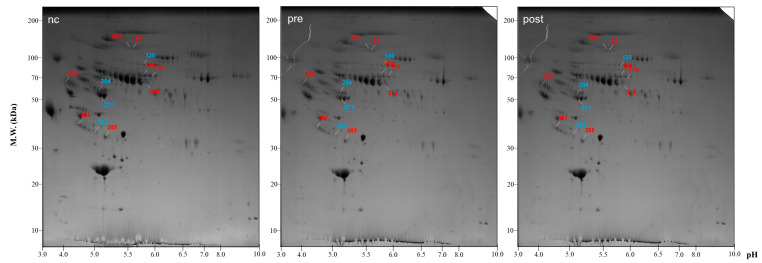
Two-dimensional gel electrophoresis map of extracted proteins from control (left row), pre-treatment (center row), and post-treatment group (right row) showing 12 differentially expressed spots. The spot number corresponded to that shown in Table 2. Red number indicates higher expression in the pre-treatment pool and blue number indicates lower expression in the pre-treatment pool. M.W.: Molecular weight, nc: normal control, pre: pre-treatment group, post: post-treatment group.

Sera collected from eight control subjects (four males and four females; mean age of 35.63 ± 5.65 years) and eight psoriasis subjects (four males and four females; mean age of 39.34 ± 4.98 years, baseline and post-treatment 52 weeks) were analyzed via 2-DE. The dissimilarity in protein expression level between the three groups was compared through 2-DE based on at least 2.5-fold difference between baseline and post-treated groups and between baseline and control groups. Dissimilarity in protein expression level was observed for twelve spots, including eight over-expressed spots and four under-expressed spots in the baseline group. Spots with red number (80, 174, 186, 203, 216, 367, 385, and 651) indicate higher expression in the pre-treatment pool with both pre/NC and pre/post ratio over 2.5 and with blue number (130, 254, 271, and 383) indicate lower expression in the pre-treatment pool with both NC/pre and post/pre ratio over 2.5 (Figure 1).

### 3.3. Identification of Differentially Expressed Proteins

The 12 spots were subjected to LC-MS/MS analysis for protein identification (Table 2). Gelsolin (protein accession number gi|121116) was determined as an under-expressed protein in the baseline group compared with both of the other two groups.

### 3.4. Measurement of Serum Levels of Gelsolin 

**Figure 2:**
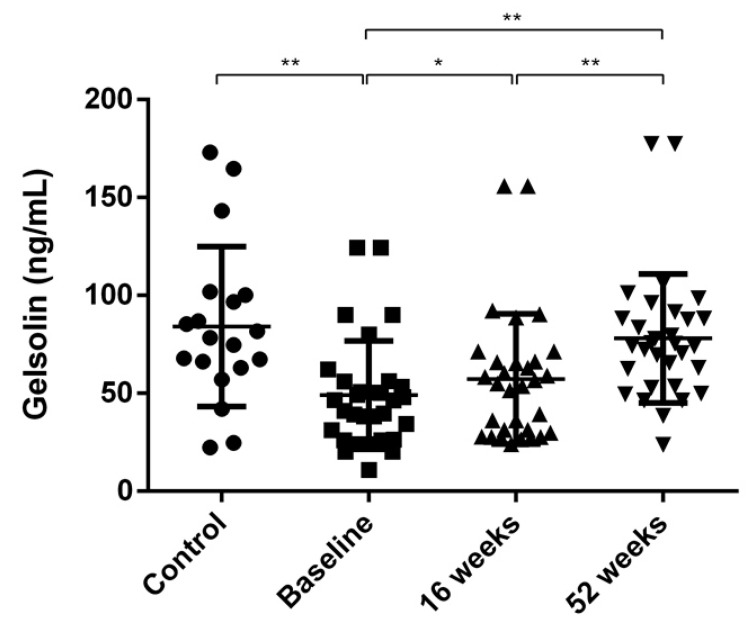
Serum gelsolin levels in control, pre-treatment (baseline), and post-treatment psoriasis groups (at 16 weeks and 52 weeks during treatment). * *p* < 0.05, ** *p* < 0.01.

Serum gelsolin levels were measured by ELISA in order to validate the resultant protein of proteomic analysis. No difference in gelsolin level was observed according to age and sex in both the control group and the psoriasis group. Serum gelsolin level was significantly lower in the baseline group (49.03 ± 27.66 ng/mL) than in the control group (84.21 ± 40.78 ng/mL, *p* < 0.01). In those with psoriasis, baseline gelsolin level was significantly lower than that at 16 weeks post-treatment (57.28 ± 33.27 ng/mL, *p* < 0.05) and 52 weeks post-treatment (78.01 ± 32.91 ng/mL, *p* < 0.01) (Figure 2).

### 3.5. Correlation between Clinical Severity Scores and Serum Gelsolin Level

**Figure 3:**
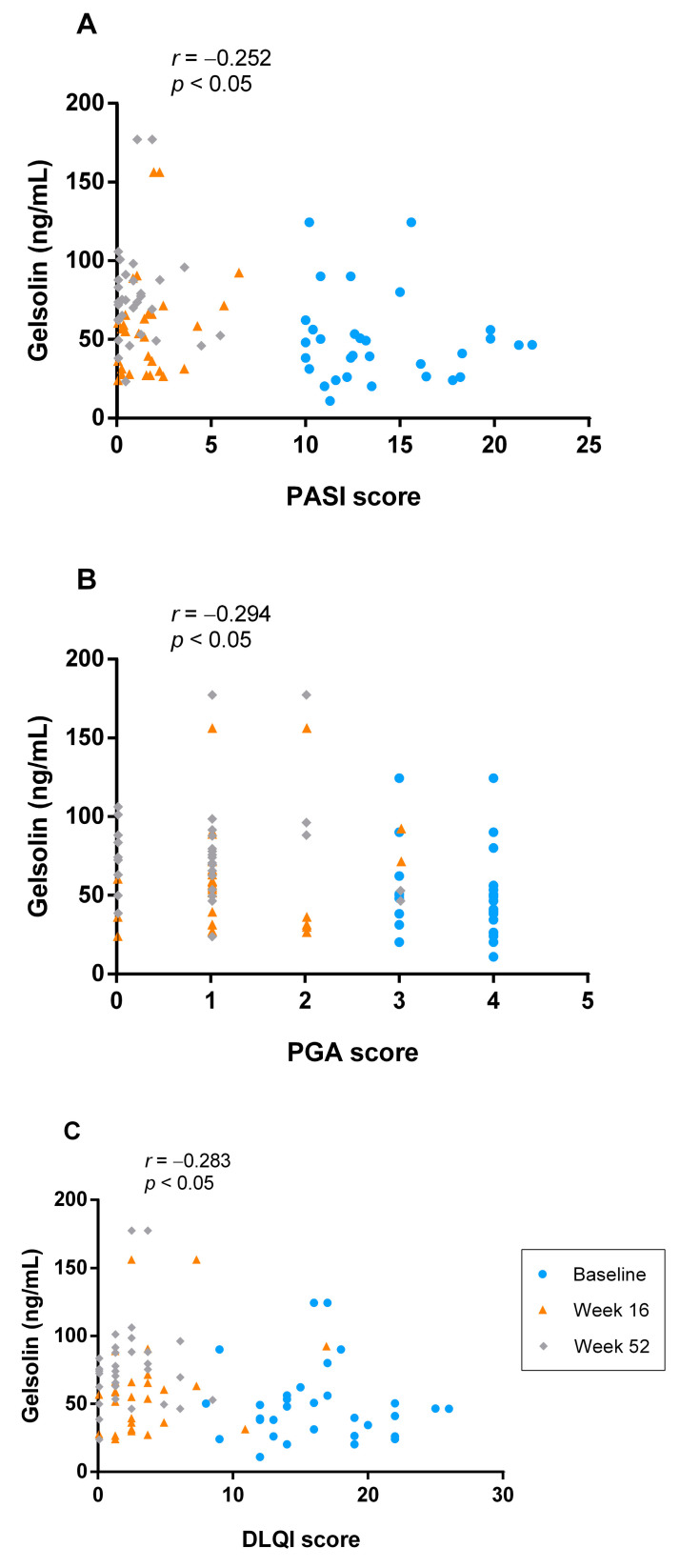
Correlation between serum gelsolin level and various clinical severity scores (**A**) PASI score (**B**) PGA score (**C**) DLQI score. DLQI: Dermatology Life Quality Index, PASI: Psoriasis Area and Severity Index, PGA: Physician Global Assessment.

We subsequently analyzed the relationship between clinical scores and gelsolin levels in psoriasis patients. Serum samples from baseline, week 16 and week 52 were pooled for analysis and the correlation was assessed by Pearson correlation coefficient using clinical scores and serum gelsolin level. At all-time points (*n* = 93), gelsolin levels showed fair negative correlation with PASI scores (*r* = −0.252, *p* < 0.05), PGA scores (*r* = −0.294, *p* < 0.05), and DLQI scores (*r* = −0.283, *p* < 0.05). (Figure 3).

The change in gelsolin level according to the type of biologic treatment was also examined. The differences in the change in gelsolin levels according to treatment with two types of biologic agents, an IL-17 inhibitor (secukinumab and ixekizumab) and an IL-23 inhibitor (guselkumab and risankizumab), was not significant (*p* = 0.06). (Supplementary Figure S1)

### 3.6. Serum Gelsolin Level in Patients with Psoriatic Arthritis and Nail Changes

**Figure 4:**
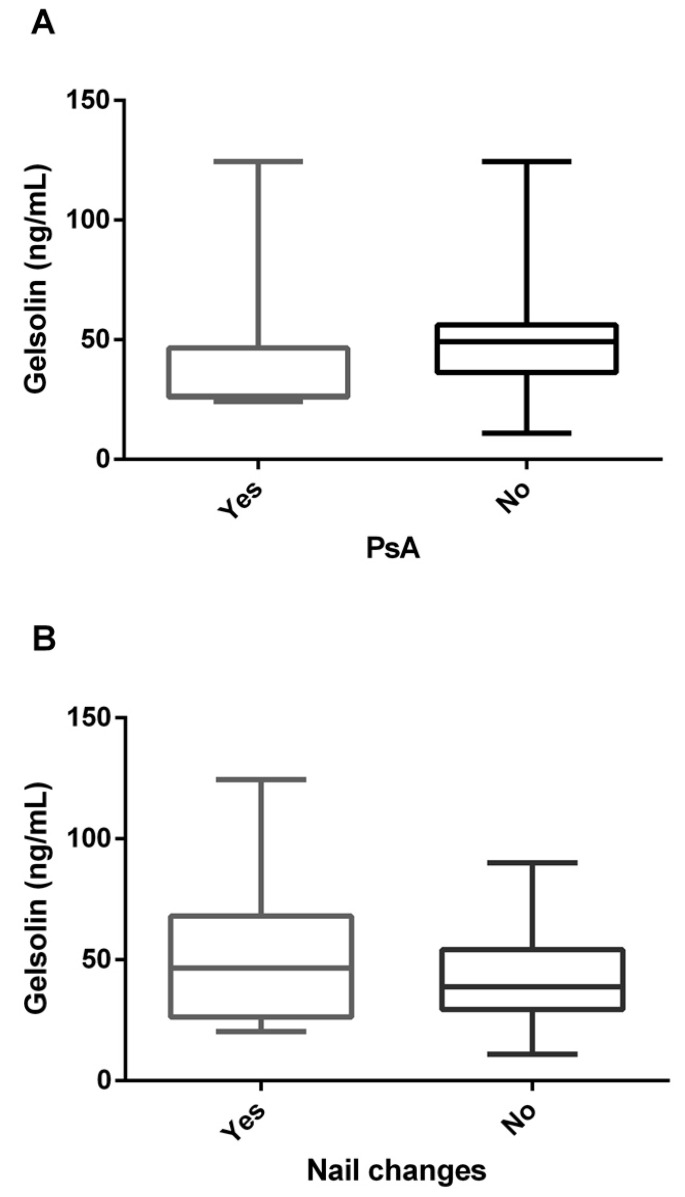
Difference in baseline gelsolin level between patients with and without (**A**) psoriatic arthritis or (**B**) nail changes. PsA: Psoriatic arthritis.

We studied whether there was a difference in baseline gelsolin level between patients with and without psoriatic arthritis (PsA) or nail changes. There were no significant differences in gelsolin level according to the presence or absence of PsA or nail changes (Figure 4).

## 4. Discussion

Psoriasis is a common inflammatory skin disorder with increased risk of comorbidities and huge socioeconomic burden [15]. This disease has different clinical phenotypes. The most common type is chronic plaque or psoriasis vulgaris. The most typical clinical picture of psoriasis shows well-demarcated, erythematous to pink colored plaques with variables size and thickness covered in silvery scales. 

Over the past 20 years, our understanding of psoriasis with complicated etiology and specific inflammatory pathways has progressively advanced, which has facilitated more accurate target rendition compared with various conventional therapies such as phototherapies and systemic medications. For moderate to severe plaque psoriasis, biologics, mostly recombinant monoclonal antibodies or receptor fusion proteins, have shown superior efficacy and safety to traditional systemic medications [3]. 

In general, treatment strategies for psoriasis should account for disease severity, accompany of underlying medical condition and presence of PsA [16]. In this context, an accurate and reasonable clinical evaluation scale is necessary to determine the severity of the disease and the best treatment options. PASI scoring system is a representative evaluation tool that has been widely applied. Additionally, PGA can be used conveniently in clinical practice. However, a downside of this clinician-oriented evaluation of disease severity is that it requires assurance in intra- or inter-observer reliability and interpretability [17]. Nevertheless, to date, these clinically validated measuring tools play a more valuable role in assessment of disease activity or severity than quantitative and qualitative measurement of biochemical or imaging markers in psoriasis, unlike other diseases. 

The definition for ‘biomarker’ may differ between researchers and institutions. The U.S. Food and Drug Administration (USFDA) introduced the term as follows; “a defined characteristic that is measured as an indicator of normal biologic processes, pathologic processes, or responses to an exposure or intervention, including therapeutic interventions”. A more detailed description of biomarker is “molecular, histologic, radiographic, or physiologic characteristics are types of biomarkers” (FDA-NIH: Biomarker-Working-Group, 2016). Accordingly, reliable biomarkers for objective evaluation of disease activity or severity could serve an essential role in reducing observatory gap associated with the use of clinical assessment tools. 

Biomarkers for psoriasis are being discovered and studied in a wide range of research fields. Depending on the purpose of discovery and clinical application, potential biomarkers of psoriasis may be subdivided into various subclasses. Specifically, biomarkers for distinguishing disease phenotypes, correlating with clinical severity and therapeutic outcome, and predicting prognosis and comorbidities, are being actively studied. Proteins responsible for the immune response in interleukin (IL)-17 pathway, management of vitamin D, systemic inflammation, and lipid metabolism are expected to serve as biomarkers in psoriasis [5,18,19,20,21]. Although many serum biomarkers related to the pathogenesis or evaluating disease activity in untreated psoriasis have been studied, relatively few studies have determined candidate biomarkers associated with altered activity or severity before and after intervention, chiefly conventional treatments or treatment with biologic agents [5,18,21,22]. 

Through this study, we tried to discover new potential serum biomarkers as well as to whether the identified biomarkers effectively reflect responses to treatment. Therefore, we established the relationship between patients’ pre- and post-treatment severity scores and identified biomarkers. We obtained results that show gelsolin could serve as a potential biomarker of clinical severity and therapeutic response in psoriasis, in accordance with comparative proteomic data analysis and identification and validation through measurement of serum gelsolin levels. 

Various studies have shown that gelsolin plays an essential role in the physiological and pathological processes in the human body [9]. Plasma gelsolin is recently considered as a diagnostic biomarker in various chronic inflammatory conditions, predictor of autoimmune diseases, and therapeutic target in neurological and neurodegenerative diseases [23]. Compared to the relatively well-known actin scavenging and clearance activity of cytosolic gelsolin, research on the role and function of gelsolin in extracellular fluids is insufficient. Blood gelsolin levels are decreased markedly in various medical conditions such as acute respiratory distress syndrome, myocardial infarction, sepsis, major trauma, malaria, liver injury, and inflammatory joint diseases [9,11,12]. 

Few studies have been conducted on the implication of gelsolin in the development and progression of psoriasis. Esawy et al. [24] reported the plasma level of gelsolin was significantly lower in psoriasis patients in comparison with controls.

In the present study, mass-spectrometry (MS)-based proteomics were used to identify gelsolin as a candidate serum biomarker. Proteomics denotes the experimental analysis of proteins in a broad sense and is one of the most advanced techniques in identifying blood biomarkers in various medical conditions. While 2-DE expedited comparative analysis of protein expression in the 1990s, subsequent MS analysis allowed precise measurement of mass and fragmentation spectra of peptides emanating from sequence-specific digestion of proteins [25]. 

Through this proteomic analysis and subsequent database search, it was possible to present a list of proteins showing differential expression levels in the pre-treatment group compared to the other two groups. After excluding proteins associated with coagulation pathway or complement system, gelsolin was finally assigned as a candidate protein for confirmative clinical validation study. Subsequent validation study with ELISA was completed initially by comparing gelsolin levels in psoriasis and control groups. We then investigated the correlation of gelsolin levels with severity scores in the group of patients with psoriasis. Comparing the biomarker levels between control and psoriasis subgroup after adjusted for age and sex, it was demonstrated that gelsolin level was lower in the pre-treatment psoriasis group than in the control and post-treatment group. This result supports the previous 2-DE result.

Next, we investigated the relationship between gelsolin and disease severity. To this end, we conducted a correlation analysis between serum gelsolin level and clinical severity using PASI, PGA and DLQI, the most widely used measurement tools in clinical fields or trials [26]. It is noteworthy that when the relationship between these clinical severity scores and serum gelsolin level was analyzed, we found a fair negative correlation. 

The role of gelsolin in various inflammatory skin diseases including psoriasis has yet to be clearly established. However, one study [24] has suggested that gelsolin is a protein marker of PsA, not only for screening PsA but also for differentiating between psoriasis and PsA. Although they failed to find correlation between blood gelsolin level and psoriasis activity, we hypothesize that in psoriasis patients, a reduction in intensity of inflammatory process through long-term treatment could be ultimately associated with an increase in gelsolin level. More in detail, in psoriasis patients, a reduction in intensity of T helper (Th)17 immune axis and resulting immune balance between Th17, Th1, Th22 and regulatory T cells through long-term treatment may have a linkage with an increase in gelsolin level. That is, although patients’ clinical scores reach PASI 90 to 100 in short period, there will be some time lag before the actual stabilization of immune axis and subsequent changes in serum levels of the biomarker. These observations may be an inducement factor for patients to require continuous treatment despite the remarkable improvement in PASI during a short treatment period. Additionally, various studies have suggested that plasma gelsolin can provoke an anti-inflammatory process by binding to pro-inflammatory mediators, which may support our finding [27,28].

Our study is to discover gelsolin as a candidate protein in psoriasis through proteomic research and then validate it as a potential novel biomarker. However, this study has three main limitations. First, we could not conduct a validation study on a large number of participants. Therefore, the number of participants in each group was insufficient to validate the change in gelsolin level according to the treatment agent. Second, although it is a concern that has always been raised in the field of biomarker research, it was ambiguous to draw a definitive conclusion regarding changes in gelsolin preceding or following progression of psoriasis. Third, in order to solidify gelsolin as a novel biomarker, another reliable validation method based on tissue analysis via immunohistochemistry of patients’ lesional and non-lesional skin is needed. A follow-up study is planned to assess the potential of this protein as a new blood biomarker by recruiting a larger number of participants. Comparative studies with known biomarkers, such as serum β-defensin, are also planned.

## 5. Conclusions

In summary, we identified gelsolin as a potential clinical biomarker of psoriasis based on 2-DE and LC-MS/MS analysis. By measuring serum gelsolin level in a case-control study, gelsolin may be considered as a biomarker with the potential to be useful in the evaluation of treatment response. Additionally, the relationship of serum gelsolin level with clinical severity was statistically established within the disease group.

## Figures and Tables

**Table 1:** Clinical characteristics of psoriasis patients.

Characteristic	Value
Age	
Min-max (med)	19–68 (39)
Mean ± SD	39.29 ± 10.32
Sex	
Male	18 (58)
Female	13 (32)
Disease duration (year)	14.48 ± 8.64
Psoriatic arthritis	
Yes	7 (22.6)
No	24 (77.4)
Nail involvement	
Yes	17 (54.8)
No	14 (45.2)
Treatment received	
MTX	3 (9.7)
Secukinumab	8 (25.8)
Ixekizumab	6 (19.4)
Guselkumab	10 (32.3)
Risankizumab	4 (12.9)
PASI score	
Week 0 (Baseline)	13.93 ± 3.63
Week 16	1.62 ± 1.55
Week 52	1.01 ± 1.33
PGA scale	-
Week 0 (Baseline)	3.70 ± 0.46
Week 16	1.20 ± 0.70
Week 52	0.94 ± 0.81
DLQI score	-
Week 0 (Baseline)	16.26 ± 4.60
Week 16	2.52 ± 1.93
Week 52	1.84 ± 1.83

Values are presented as number (%) or mean ± standard deviation. DLQI: Dermatology Life Quality Index, MTX: methotrexate. PASI: Psoriasis Area and Severity Index, PGA: Physician’s Global Assessment.

**Table 2:** Identified protein showing differential expressions in 2-DE image analysis.

Group No.	Protein Accession Number	Protein Name	M_r_	pI	Sequence Coverage (%)	Matches Peptide Number
80	gi|179665	Complement component C3	88,585	6.02	11	14
130	gi|121116	Gelsolin	86,043	5.90	8	7
174	gi|190500	C4b-binding protein alpha chain	69,042	7.15	7	7
186	gi|23200172	Chain A, Heparin Cofactor Ii	55,096	6.26	11	11
203	gi|443345	Chain A, Alpha 1-antichymotrypsin	40,690	5.09	37	35
216	gi|112874	Alpha-1-antichymotrypsin	47,792	5.33	11	8
254	gi|113880	Angiotensinogen	53,406	5.87	21	15
271	gi|181482	Serum vitamin D-binding protein precursor	54,612	5.40	22	51
367	gi|116533	Clusterin	53,031	5.89	18	11
383	gi|115896	Carboxypeptidase N catalytic chain	52,538	6.86	10	6
385	gi|230651	Chain A, transthyretin precursor	13,809	5.55	33	35
651	gi|179665	Complement component C3	188,585	6.02	12	30

Mr: molecular mass, pI: isoelectric point.

## Data Availability

The datasets generated during and/or analysed during the current study are available from the corresponding author on reasonable request.

## Supplementary Materials

The following supporting information can be downloaded at: https://www.mdpi.com/article/10.3390/jcm12051801/s1, Figure S1: Changes in gelsolin levels according to treatment with two types of biologic agents. IL: interleukin, mAbs: monoclonal antibodies.
