# Supplementary material for: Gelsolin as a Potential Clinical Biomarker in Psoriasis Vulgaris
Source: J Clin Med. 2023 Feb 23;12(5):1801. doi: 10.3390/jcm12051801 (PMC10003618; doi:10.3390/jcm12051801)
Supplement: Supplementary file 1 [file jcm-12-01801-s001.zip › jcm-2119535-supplementary.pdf]

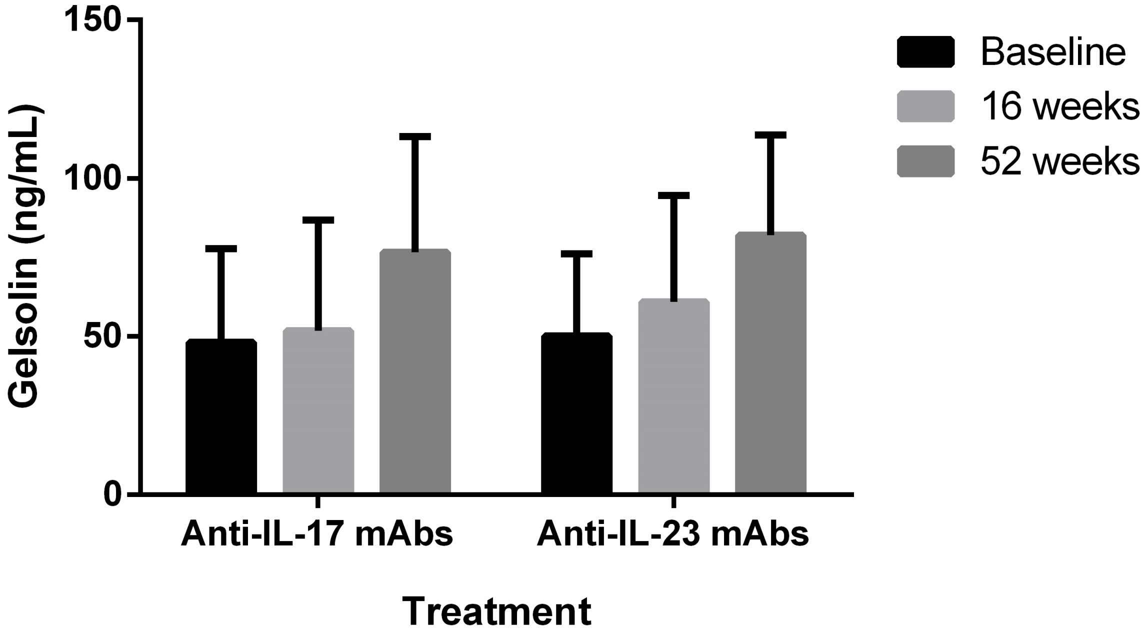

Figure S1: Changes in gelsolin levels according to treatment with two types of biologic agents. IL: interleukin, mAbs: monoclonal antibodies.
